# Time Is Not More Abstract Than Space in Sound

**DOI:** 10.3389/fpsyg.2019.00048

**Published:** 2019-02-01

**Authors:** Alexander Kranjec, Matthew Lehet, Adam J. Woods, Anjan Chatterjee

**Affiliations:** ^1^Department of Psychology, Duquesne University, Pittsburgh, PA, United States; ^2^Center for the Neural Basis of Cognition, Carnegie Mellon University, Pittsburgh, PA, United States; ^3^Department of Psychology, Carnegie Mellon University, Pittsburgh, PA, United States; ^4^Cognitive Aging and Memory Clinical Translational Research Program, Institute on Aging, University of Florida, Gainesville, FL, United States; ^5^Department of Aging and Geriatric Research, University of Florida, Gainesville, FL, United States; ^6^Department of Neurology, University of Pennsylvania, Philadelphia, PA, United States

**Keywords:** space perception, time perception, pitch perception, embodied cognition, conceptual metaphor theory

## Abstract

Time is talked about in terms of space more frequently than the other way around. Some have suggested that this asymmetry runs deeper than language. The idea that we think about abstract domains (like time) in terms of relatively more concrete domains (like space) but not *vice versa* can be traced to Conceptual Metaphor Theory. This theoretical account has some empirical support. Previous experiments suggest an embodied basis for space-time asymmetries that runs deeper than language. However, these studies frequently involve verbal and/or visual stimuli. Because vision makes a privileged contribution to spatial processing it is unclear whether these results speak to a general asymmetry between time and space based on each domain’s general level of relative abstractness, or reflect modality-specific effects. The present study was motivated by this uncertainty and what appears to be audition’s privileged contribution to temporal processing. In Experiment 1, using an auditory perceptual task, temporal duration and spatial displacement were shown to be mutually contagious. Irrelevant temporal information influenced spatial judgments and *vice versa* with a larger effect of time on space. Experiment 2 examined the mutual effects of space, time, and *pitch*. Pitch was investigated because it is a fundamental characteristic of sound perception. It was reasoned that if space is indeed less relevant to audition than time, then spatial distance judgments should be *more* easily contaminated by variations in auditory frequency, while variations in distance should be *less* effective in contaminating pitch perception. While time and pitch were shown to be mutually contagious in Experiment 2, irrelevant variation in auditory frequency affected estimates of spatial distance while variations in spatial distance did not affect pitch judgments. Results overall suggest that the perceptual asymmetry between spatial and temporal domains does not necessarily generalize across modalities, and that time is not generally more abstract than space.

## Introduction

*Time* is frequently talked about using the language of *space* (Clark, 1973; Haspelmath, 1997; Tenbrink, 2007). A meeting can be *long* or *short*, and *occupy a place* that is either *behind* or *in front* of us *in* time. Space is used to talk about time not only frequently but also meaningfully. We talk about temporal extent or *duration* in terms of distance (e.g., a short time), and the *past and future* in egocentric locational terms (e.g., the past is behind us). These ways of talking and thinking about space and time are thought to reflect something about how we experience these domains together. We may talk about duration in terms of *length* because it takes more time to visually scan or travel through a more extended space, and the past as *behind* because as we walk forward, objects we pass begin to occupy the unseen space behind our bodies becoming accessible only to memory and part of a temporal past. Experimental studies support the idea that the ways in which we experience space play a role in structuring the semantics of time (Boroditsky, 2000, 2001; Boroditsky and Ramscar, 2002; Matlock et al., 2005; Nunez and Sweetser, 2006; Nunez et al., 2006; Torralbo et al., 2006; Casasanto and Boroditsky, 2008; Kranjec et al., 2010; Miles et al., 2010; Kranjec and McDonough, 2011). See Nunez and Cooperrider (2013) for a recent review of experimental research, and Evans (2013) for a perspective from cognitive linguistics.

In semantics, time–space relations are relatively asymmetrical. Not only is time lexicalized in spatial terms much more frequently than *vice versa*, but in many ways time must be conceptualized using the language of space, whereas the opposite is not true (Jackendoff, 1983; Casasanto and Boroditsky, 2008). [However, see Tenbrink (2007) for a discussion of how such asymmetric mapping relations do not necessarily apply to discourse, and a general perspective on time–space relations that is highly compatible with the one presented in the current study.] These linguistic patterns have been interpreted to suggest a deeper conceptual organization. According to *conceptual metaphor theory* (Lakoff and Johnson, 1999) we think about relatively abstract *target* domains (like time) in terms of more concrete *source* domains (like space). This basic organizational principle is purported to serve the functional role of making more abstract concepts easier to talk *and think* about. It is argued that we depend on such a hierarchy because, for example, we can directly see and touch things “in space” in a way that we cannot “in time.” This suggests that thinking about time in terms of space runs cognitively deep, and reflects a mental organization more fundamental than that observed at the relatively superficial level of semantics.

In a widely cited paper, Casasanto and Boroditsky (2008) sought strong experimental evidence for this theoretical organizational principle. Specifically, they wanted to know if the asymmetry of space-time metaphors in language predicted a similar asymmetry in perception. They reasoned that low-level perceptual biases demonstrating concordant asymmetry with patterns found in language would provide strong evidence that temporal representations are grounded in more concrete spatial representations.

In their study, participants viewed growing or static lines one at a time on a computer screen. Lines could be of nine durations crossed with nine displacement sizes to produce 81 unique stimuli. After the presentation of each line, participants were randomly prompted to either reproduce a line’s spatial extent (by dragging a mouse) or a line’s duration (by clicking a mouse). Each line was presented twice: once in each kind of reproduction trial (i.e., displacement or duration estimation).

They found that the remembered size of a line in space concordantly modulated recall for its duration, but not *vice versa*. That is (spatially), longer lines were remembered as being presented for longer times, but lines of greater durations were not remembered as having greater spatial extent. The results were consistent with the idea that asymmetrical patterns of space-time mappings in language are preserved further down at the level of perception. They concluded, “these findings provide evidence that the metaphorical relationship between space and time observed in language also exists in our more basic representations of distance and duration” (p. 592). Similar results reporting asymmetrical effects have been found with children (Casasanto et al., 2010) but not with monkeys (Merritt et al., 2010) or pigeons (De Corte et al., 2017).

That humans use space to think about time is now widely acknowledged. The idea that time is fundamentally more abstract (and less accessible to the senses) than space may be regarded as a prerequisite for this relation. However, there are still reasons to question this general organizational principle constraining “links between the abstract domain of time and the relatively concrete domain of space” (Casasanto, 2010, p. 455). At least, there might be some misunderstanding about what it means for time to be more abstract than space.

First, neural data supporting the idea that our temporal concepts are grounded in embodied spatial representations is scarce, partly because it is not entirely clear what an embodied spatial representation *is* in the first place (Kranjec and Chatterjee, 2010). Furthermore, recent fMRI evidence suggests that temporal and spatial concepts do not necessarily have privileged relations in the brain too. In an experiment (Kranjec et al., 2012) designed to look for functional architecture shared among basic abstract semantic categories (space, time, and causality), brain areas associated with the spatial extent of simple events had little overlap with those associated with their temporal duration. By focusing on space, embodied theories have neglected to investigate temporal conceptual grounding in neural systems that instantiate time perception in the body.

Another issue concerns what is meant by “concrete” and “abstract” in the Conceptual Metaphor Theory literature. In defining the distinction between concrete and abstract thought, Lakoff (2014) writes:

Our current theory begins with a basic observation: The division between concrete and abstract thought is based on what can be observed from the outside. Physical entities, properties, and activities are “concrete.” What is not visible is called “abstract:” emotions, purposes, ideas, and understandings of other non-visible things (freedom, time, social organization, systems of thought, and so on).”

Or according to a more recent description according to Mental Metaphor Theory:

That is, people often think in “mental metaphors”… point-to-point mappings between non-linguistic representations in a “source domain” (e.g., SPACE) and a “target domain” (e.g., TIME) that is typically more abstract (i.e., hard to perceive) or abstruse (i.e., hard to understand; Lakoff and Johnson, 1980), which support inferences in the target domain (Casasanto, 2017, p.47).

While there is little agreement among philosophers regarding what counts as an abstract or concrete concept (Rosen, 2018), generally speaking concrete kinds of representations are those that refer to physical objects that can be experienced directly through the senses. Regardless, behavioral studies in this area of research frequently rely on visual tasks and, perhaps more controversially, there is a tendency to conflate “space” with what could be more accurately described as the “visuospatial.” This makes it unclear whether previously observed behavioral asymmetries between time and space reflect (1) very general differences in how humans process the abstract domains of space vs. time [E.g., “Aspects of time are often said to be more “abstract” than their spatial analogs because we can perceive the spatial, but we can only imagine the temporal (Casasanto and Boroditsky, 2008, p. 580)] or (2) a less general, modality-specific contribution of visual representations in humans. That is, perhaps space-time asymmetries discussed in previous behavioral studies can be better understood in terms of visual biases and do not directly reflect how differences in the relative abstractness of space vs. time may serve as a general organizing principle in human cognition. In fact, perceptual asymmetries between space and time may be better understood in terms of their *relevance* with respect to a particular modality more than their imagined placement on a concrete-abstract continuum.

To distinguish between these two alternatives, the present study directly probes time–space relations in the auditory domain. Audition was selected because there are intuitive reasons to think that those time–space asymmetries observed in vision might actually be reversed in sound. Phenomenologically, time, more than space, seems to be an intimate part of our auditory experience. [But see (Shamma, 2001) for a dissenting view]. For example, whereas spatial relations and visual objects tend to be persistent, sound, like time, is relatively transient (Galton, 2011). Temporal information is more meaningful and/or salient in common forms of experience grounded in sound perception (e.g., music and speech). In the context of music, “when” a sound occurs matters much more than “where” it occurs. There are neuropsychological reasons too. While the retina preserves analog spatial relations in early representations, the cochlea does not (Ratliff and Hartline, 1974; Moore, 1977). That is, the pattern of activation on the sensory surface of the eye is representative of the relative spatial relations among visual objects in an array, and these relations are further preserved topologically in the cortex. In the auditory system spatial relations between auditory objects are computed in the cortex, achieved via a temporal mechanism (interaural time difference); there is no direct representation of these spatial relations preserved on the primary sensory surface of the cochlea. For these reasons, sound localization is less precise than object localization in vision (Kubovy, 1988). In speech, the ability to perceive differences in voice onset time is critical for discriminating between phonological categories (Blumstein et al., 1977).

Temporal relations, as compared to spatial ones, appear to be more relevant to hearing as indicated by the relatively concrete manner that temporal information is represented, processed, experienced, and embodied in the auditory system. While one might argue that *relations* between sound and time are relatively *more concrete* (i.e., more directly accessible to the senses) than relations between sound and space, perhaps it is more accurate to say that time is more modality-relevant than space in audition. While the difference between *concreteness* and *modality-relevance* may in part be a historical-philosophical distinction, the present research addresses some issues raised by how concreteness is frequently discussed in the literature with a task closely following Casasanto and Boroditsky (2008) but using auditory instead of visual stimuli. It asks: are the kinds of space-time asymmetries observed in previous studies using visual stimuli also observed in a purely auditory task?

## Experiment 1

### Methods

#### Ethics Statement

This study was approved by the Institutional Review Board at the University of Pennsylvania. Written informed consent was obtained from all participants.

#### Participants

Twenty members of the University of Pennsylvania community participated for payment. All participants were right-handed, native English speakers, and between 18 and 26 years of age.

#### Procedure and Experimental Design

The participants were equipped with headphones and seated at a computer for a self-paced experiment. Participants initiated the beginning of each new trial and the start of each within-trial component. Each trial consisted of two sounds, a *target sound* followed by a *playback sound*. In the first part of each trial, the target sound was presented, and participants were instructed to attend to both spatial and temporal aspects of the stimulus. Target sounds consisted of bursts of white noise that changed in location relative to a participant’s head position across time. White noise bursts were of nine durations (lasting between 1000 and 5000 ms with 500 ms increments) and nine distances (moving between 0.5 and 4.5 m in increments of 0.5 m). All durations and distances were crossed to create 81 distinct target sounds. The initial location of the target sound was an average of 2.75 m to the left or right of the listener with a jitter of between 0.1 and 0.5 m. The plane of movement was 1 m in front of the listener. Starting locations on the right indicated leftward moving trials and starting locations on the left indicated rightward moving trials. Starting locations were randomly assigned to stimuli with an even number of right and leftward moving trials. Stimuli were created using MATLAB and played using the OpenAL library provided with Psychophysics Toolbox extensions (Brainard, 1997). The OpenAL library is designed to model sounds moving in virtual metric space for a listener wearing headphones using head related transfer functions (HRTFs).

After attending to the target sound, participants were prompted to reproduce either the sound’s duration or distance and then instructed to press the spacebar to begin the playback sound. In this second part of each trial, the playback sound provided the medium for the participant’s response. The playback sound began in the final location of the preceding target sound and moved in the reverse direction. So, if a target sound moved rightward, the playback sound moved leftward, and *vice versa*. On *distance* trials, participants were instructed to respond when the playback sound reached the start location of the target sound, thereby reproducing the distance from head to start point. In this manner, the participant’s head provided a fixed reference point for judging distance. On *duration* trials, participants were instructed to respond when the playback sound duration was equal to the target sound duration. The playback sound lasted for a fixed 8500 ms and moved 3.5 m past the starting location of the target sound or until the participant responded. The playback sounds were designed in such a manner as to allow participants the possibility to both overshoot and undershoot their estimates. Participants heard each target sound in both duration and distance conditions (within-subject design) for a total of 162 trials.

### Results

**FIGURE 1:**
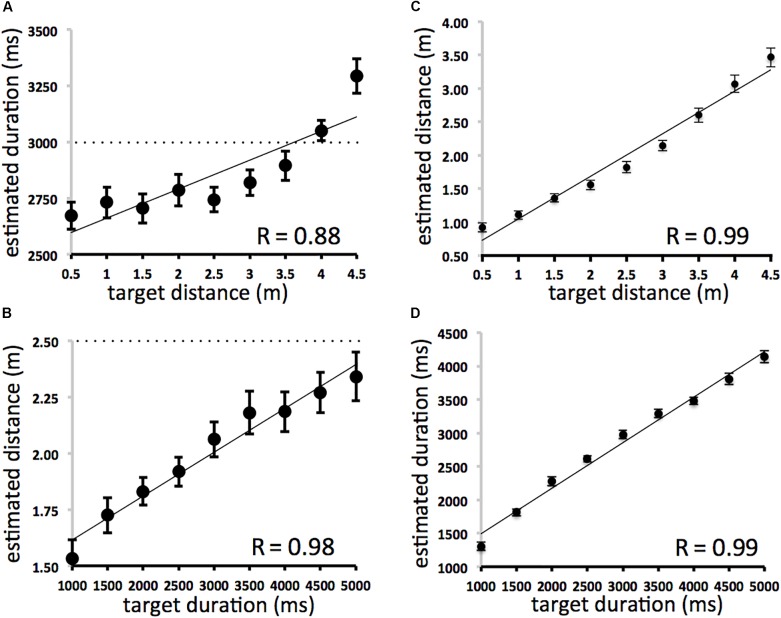
**(A–D)** Averaged duration and spatial displacement estimates. Scatterplots **(A)** (SPACE→TIME) and **(B)** (TIME→SPACE) on left depict between domain effects. The dotted lines represent the line predicted by perfect performance. All space and time intervals were fully crossed. The average of all nine duration intervals is 3000 ms at each displacement length **(A)** and the average of all nine displacement intervals is 2.5 m at each duration **(B)**. Scatterplots **(C)** (SPACE→SPACE) and **(D)** (TIME→TIME) on the right depict within domain effects. Error bars refer to standard error of the mean.

The results (Figure 1) demonstrate that actual spatial displacement affected estimates of duration (Figure 1B: *y* = 128.97× + 2532.8, *r* = 0.878, *df* = 7, *p* < 0.01) *and* that actual durations affected estimates of spatial displacement (Figure 1A: *y* = 0.0002× + 1.4208, *r* = 0.982, *df* = 7, *p* < 0.01). On *duration* trials, for stimuli of the same average displacement (2.5 m) sounds of shorter durations were judged to be shorter in length, and sounds of longer durations were judged to be longer in length. On *distance* trials, for stimuli of the same average duration (3000 ms), sounds shorter in length were judged to be of shorter duration, and sounds longer in length were judged to be of longer duration. Space and time were mutually contagious in that irrelevant information in the task-irrelevant domain affected participants’ estimates of both duration and spatial displacement. Compatible effects were found using multiple regression analyses. Distance was significantly correlated with duration judgments when variance associated with actual duration was removed [ρ*r*(81) = 0.64; *df* = 80, *p* < 0.01]. Duration was significantly correlated with distance judgments even when variance associated with each trial’s actual distance was removed [ρ*r*(81) = 0.81; *df* = 80, *p* < 0.01] (Sample *N* = 81 [nine space and nine time intervals fully crossed]). There was no effect of direction (left-moving vs. right moving trials).

Participants’ overall estimates of duration and displacement were very accurate. The effects of actual displacement on estimated displacement (Figure 1C: *y* = 0.6374× + 0.4115, *r* = 0.99, *df* = 7, *p* < 0.001) and actual duration on estimated duration (Figure 1D: *y* = 0.6805× + 813.64, *r* = 0.99, *df* = 7, *p* < 0.001) were also very similar to each other and to analogous analyses of accuracy in Casasanto and Boroditsky (2008). This suggests that participants were approximately equal in accuracy when making duration and distance judgments within the present experiment and between comparable experiments using auditory and visual stimuli. It also suggests that spatial and temporal changes are no more or less “hard to perceive” (Casasanto, 2017) in the approach used here.

**FIGURE 2:**
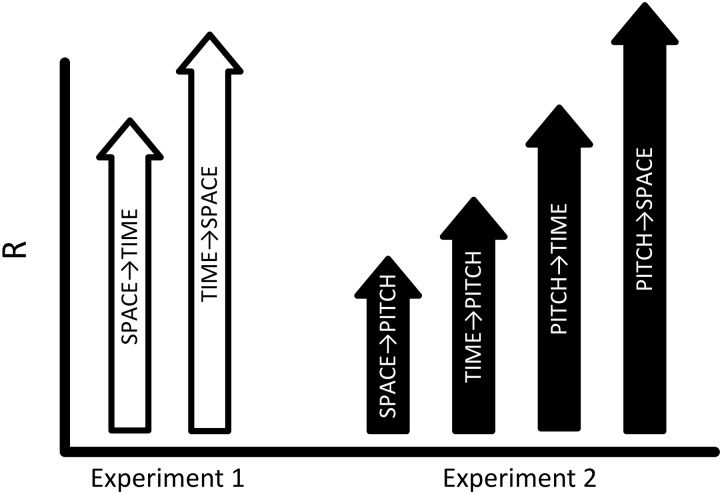
Space, Time, and Pitch in Sound. Experiment 1 (white arrows) found that time and space are mutually contagious, with a larger effect of time on space (SPACE→TIME < TIME→SPACE; Rs, 0.88 < 0.98). Experiment 2 investigates relations between space, time, and pitch. If PITCH is more modality-relevant to auditory perception than TIME, and TIME is more relevant than SPACE then the pattern of results represented by the black arrows is predicted in Experiment 2. That is, Rs for each condition are expected to follow a pattern where the effects of SPACE→PITCH < TIME→PITCH < PITCH→TIME < PITCH→SPACE.

The effect of duration on displacement was significantly greater than the effect of displacement on duration (See Figure 2: Fisher r-to-z transformation, difference of correlations = 0.104; *z* = 1.7 one-tailed, *p* < 0.05). However, some caution should be taken when interpreting this result. It is unclear to us whether differences in perceptual judgments between domains can be directly compared at such a fine grain when arbitrarily defined scales, intervals, and ranges (e.g., in seconds and meters) are used to define temporal and spatial aspects of the stimuli. This is a concern even though spatial and temporal judgments focused on identical stimuli. It is possible that other scaled relations could yield different patterns of results.

### Experiment 1 Discussion

While strong claims about deeply embodied asymmetrical relations between space and time in the auditory domain may be premature, Experiment 1 found a significant pattern of time–space asymmetry in the auditory domain. This asymmetry is predicted by the temporal quality of auditory processing and runs in the opposite direction of the asymmetry found in the visual domain as predicted by Conceptual Metaphor Theory and patterns of language use (Casasanto and Boroditsky, 2008). The results suggest that the spatial nature of vision more than space *per se* explains results of previous studies. So while one may suggest that time is relatively “concrete” as compared to space in sound (using the terms provided by Conceptual Metaphor Theory) it may be more useful to think about time as more “relevant” in the auditory modality. Either way, temporal representations may be more directly embodied or salient in audition as compared to spatial representations.

While the results of Experiment 1 are suggestive of a perceptual asymmetry running opposite to that observed in the visual domain, broader claims regarding any deep asymmetry between time and space in the auditory domain are premature. Although the results from Experiment 1 suggest that “in sound,” time appears to influence judgments of spatial displacement more than *vice versa*, these results may not generalize to other aspects of auditory phenomena. To make stronger claims about the relevance of space and time in the auditory domain, Experiment 2 extends the current approach, testing the manner in which representations of space and time contaminate an aspect of auditory perception that is itself directly represented by the nervous system. Whereas space and time are abstract facets of any perceptual modality, *pitch* is a fundamental attribute of hearing; analogous to color, or brightness in vision (Boring, 1933; Marks, 2004).

## Experiment 2

To further probe the relative effects of space and time in the auditory modality, Experiment 2 examines the mutual effects of space, time, and *pitch*, a uniquely auditory attribute. The perception of pitch makes possible the processing of melody in music, and prosody in speech. Defined as the perceived frequency or “repetition rate of an acoustic waveform” (Oxenham, 2012) pitch is, together with loudness and timbre, one of three basic auditory sensations. Current theories suggest that properties of the physical stimulus and the physiological mechanisms for transduction and neural representation, in addition to prior experience, all play a significant role in pitch perception. This most likely involves both temporal and place coding throughout the auditory system. When sound enters the cochlea, the distinct frequencies that make up an acoustic waveform activate tuned neural sites arranged along its membrane in a spatially analog manner. Such *tonotopic*, “rate-place” (or time–space) mapping is preserved in the auditory processing system as far as the primary auditory cortex. [See Oxenham (2012) for a review]. As such, pitch perception involves the representation of both spatial and temporal information at multiple levels of processing. The centrality and salience of pitch perception in auditory experience, and its fundamental spatiotemporality make it an ideal domain for further testing hypotheses supported by the results of Experiment 1.

Another reason pitch is an interesting domain to interrogate in the present study is that across many languages we talk about pitch in terms of space (e.g., tones can be described as “high” or “low”). Based on Conceptual Metaphor Theory, pitch as the target domain in such a mapping is assumed to be *more abstract* as compared to space, the source domain. According to such a formulation, we talk about pitch in terms of space because spatial relations are easier to conceptualize. However, with respect to the approach taken here, pitch as a fundamental attribute of auditory perception with a specific sensory mechanism devoted to its representation, can be reasonably conceptualized as *more modality-relevant* to both space and time in the auditory modality. In this manner, the inclusion of pitch allows for competing predictions for Conceptual Metaphor Theory and the kind of modality-relevant explanation introduced in the current study. If we talk about pitch in terms of space because space is relatively “less abstract,” then changes in spatial distance should contaminate judgments of pitch more than *vice-versa*. However, if modality-relevance determines the strength of contamination effects, then the opposite pattern of results should be observed. In general, if a representational domain (space, time, and pitch) is more relevant and/or directly perceivable in a particular modality (audition) then it should be more effective in contaminating less relevant domains and less vulnerable to contamination by others.

Based on the results of Experiment 1, we reasoned that in comparing space and time, spatial distance, as representative of a less modality-relevant domain, should be *less* effective (as compared to duration) in contaminating the perception of pitch in a procedure using purely auditory stimuli. We can further predict a range of transitive effects based on the relative degree of modality-relevance for space, time, and pitch. If the relations of modality-relevance are such that: space < time (based on the argument presented, and the results of Experiment 1) and space < time < pitch (based on pitch being a fundamental attribute of audition with a unique physiological mechanism for sensory transduction), then the expected results should follow the general pattern displayed in Figure 2.

### Methods

#### Participants

Forty-two members of the University of Pennsylvania community participated for payment. All participants were right-handed, native English speakers, and between 18 and 26 years of age. Twenty participants performed Experiment 2A. Twenty-two distinct participants performed Experiment 2B. Data from two of these participants were excluded from the final analyses because their reaction times across conditions were greater than two standard deviations from the mean.

#### General Procedure and Design

The general procedure and design of Experiment 2 was identical to that of Experiment 1. Participants were equipped with headphones and seated at a computer for a self-paced experiment. Participants initiated the beginning of each new trial and the start of each within-trial component. Each trial consisted of two sounds, a *target sound* followed by a *playback sound*. In the first part of each trial, the target sound was presented, and participants were instructed to attend to *either* the duration and pitch of the stimulus (Experiment 2A) *or* the distance and pitch of the stimulus (Experiment 2B). After attending to the target sound, participants were informed of the trial type and instructed to press the spacebar to begin the playback sound. The playback sound provided the medium for the participant to reproduce either the spatial displacement, duration, or pitch depending on the experiment and trial type. As in Experiment 1, all stimuli were created using MATLAB and played using the OpenAL library provided with Psychophysics Toolbox extensions (Brainard, 1997).

### Experiment 2A: Space and Pitch

In Experiment 2A participants (*N* = 20) were instructed to attend to both the *distance and pitch* of the stimulus. Target sounds were of nine distances [moving between 0.5 and 4.5 m in increments of 0.5m] (as in Experiment 1), and nine frequencies ranging between 150 and 1350 Hz in increments of 150 Hz, all crossed to create 81 discrete stimuli. The initial location of the target sound was an average of 2.75 m to the left or right of the listener with a jitter of between 0.1 and 0.5 m. Starting locations on the right indicated leftward moving trials and starting locations on the left indicated rightward moving trials. Starting locations were randomly assigned to stimuli with an even number of right and leftward moving trials. The plane of movement was one meter in front of the listener. Stimuli were created using MATLAB and played using the OpenAL library provided with Psychophysics Toolbox extensions (Brainard, 1997).

After attending to the target sound, participants in Experiment 2A were informed of the trial type (distance or pitch) and instructed to press the spacebar to begin the playback sound. The playback sound provided the medium for the participant’s response. The playback sound began in the final spatial location and frequency endpoint of the preceding target sound and moved in the reverse direction (both in terms of space and pitch). Directionality in space (left to right or right to left) and pitch (high to low or low to high) was randomized across all trials. On *distance* trials, participants were instructed to respond when the playback sound reached the start location of the target sound. In this manner, the participant’s head provided a fixed reference point for judging distance. On *pitch* trials, participants were instructed to respond when the playback sound spanned the target sound’s frequency range.

### Experiment 2B: Time and Pitch

The procedure for Experiment 2B was identical to that in 2A but with *duration* replacing distance as a domain of interest. In Experiment 2B, when the target sound was presented, participants (*N* = 22) were instructed to attend to both the *duration and pitch* of the stimulus. The target sound in Experiment 2B was a sound consisting of a variable and continuous range of frequencies presented over a variable period of time in both ears. Target sounds were of nine durations (lasting between 1000 and 5000 ms with 500 ms increments as in Experiment 1) and nine frequencies ranging between 150 and 1350 Hz in increments of 150 Hz (as in Experiment 2A). All durations and frequencies were crossed to create 81 distinct target sounds. Each discrete stimulus was used twice, once in the duration condition and once in the pitch condition. The initial frequency of the target sound began within the higher (2250 Hz) or lower (990 Hz) ends of the audible range of speech with a randomized jitter between 1 and 50 Hz. Frequency endpoints were determined by varying the number of frequency increments the sound moved through across trials. Frequency “direction” (high to low, or low to high) was random across trials.

After attending to the target sound, participants in Experiment 2A were informed of the trial type (duration or pitch) and instructed to press the spacebar to begin the playback sound. The playback sound provided the medium for the participant’s response. It presented the same frequency ranges in the opposite direction, starting at the frequency endpoint of the target sound and moving toward the start point and lasted for a maximum of 8.5 s or until the participant ended the trial by responding. On *duration* trials, participants were instructed to respond when the playback sound duration was equal to the target sound duration. On *pitch* trials, participants were instructed to respond when the playback sound span equaled that of the target sound’s frequency range. For all trials, there were at least five additional frequency increments and seven additional duration increments within the playback sound to allow participants the possibility to both overshoot and undershoot their estimates. Data for both duration and frequency judgments were collected regardless of condition.

### Results: Experiments 2A and 2B

**FIGURE 3:**
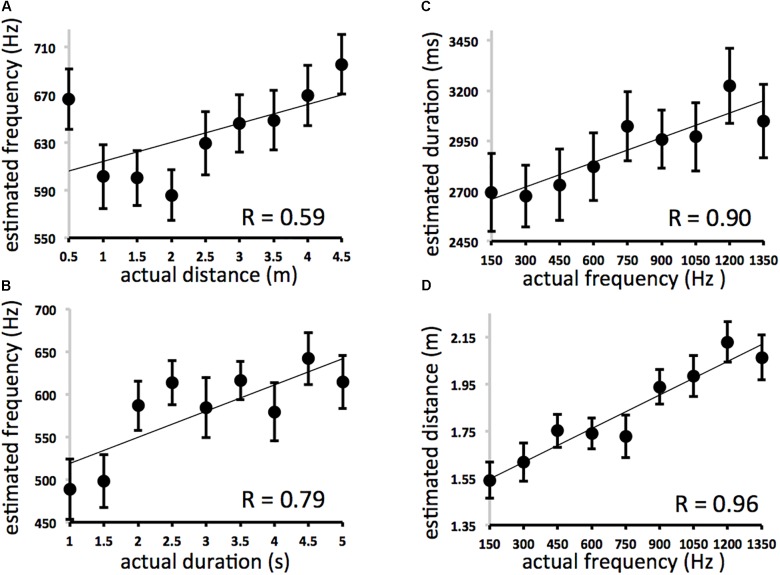
Results for Experiments 2A and 2B. Because all nine intervals used for each domain were fully crossed in Experiment 2, the expected average for estimates across all participants for a particular trial type (distance, duration, or frequency estimation; *y*-axis) can be described as the average of all nine interval values for that domain presented at each interval of the irrelevant distractor domain (actual frequency, distance, or duration; *x*-axis). If the irrelevant domain on *x* exerted no influence on estimation for *y* one would expect a horizontal line. Deviation from that horizontal represents cross-domain interference. **(A)** Effect of distance on frequency estimates (expected = 750 Hz at each interval of actual distance). **(B)** Effect of duration on frequency estimates (expected = 750 Hz at each interval of actual duration). Error bars refer to standard error of the mean. **(C)** Effect of frequency on duration estimates (expected = 3000 ms at each interval of actual frequency). **(D)** Effect of frequency on distance estimates (expected = 2.5 m at each interval of actual frequency).

**FIGURE 4:**
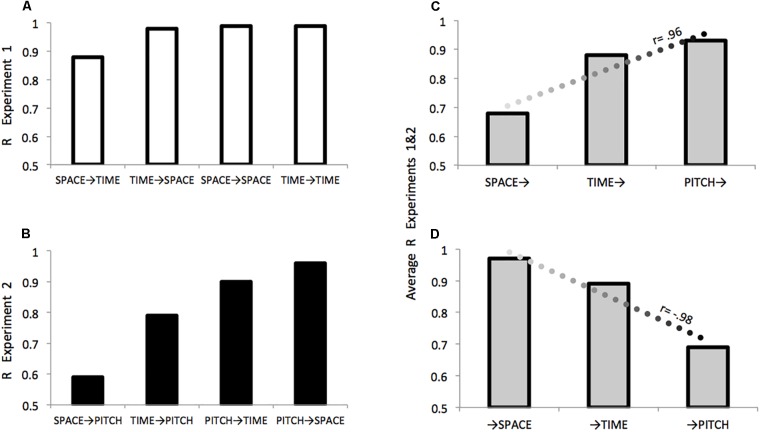
Comparing effects within and between Experiments 1 and 2. **(A)** Effects of: Displacement on Duration (SPACE→TIME); Duration on Displacement (TIME→SPACE); Displacement on Displacement (SPACE→SPACE); Duration on Duration (TIME→TIME). **(B)** Effects of: Duration on Frequency (TIME→PITCH); Duration on Frequency (TIME→PITCH); Frequency on Duration (PITCH→TIME); Frequency on Displacement (PITCH→SPACE). For both Experiments 1 and 2 *R* values are consistent with predictions depicted in Figure 2. For each domain across Experiments 1 and 2, **(C)** describes the relation between increasing relevance in the auditory modality and a particular domain’s (*X*→) effectiveness in modulating other domains as an *agent* of contamination, **(D)** describes the relation between increasing relevance in the auditory modality and the extent a particular domain (→*X*) is modulated by other domains as a *patient* sensitive to contamination.

Between Experiments 2A and 2B there are four main correlations to consider. They describe the effects of frequency on (A) distance estimates (PITCH→SPACE) and (B) duration estimates (PITCH→TIME) and the effects of (C) distance and (D) duration on frequency estimates (SPACE→PITCH and TIME→PITCH, respectively). These results are displayed in Figure 3. A comparison of *r* values between conditions/experiments is depicted in Figure 4.

The effect of distance on frequency estimation (Figure 3A) was not significant (*y* = 15.955× + 598.21, *r* = 0.593, *df* = 7, *p* = 0.09), while actual duration affected estimates of frequency (Figure 3B) (*y* = 30.7× + 488.22, *r* = 0.793, *df* = 7, *p* = 0.01). Actual frequency affected estimates of duration (Figure 3C) (*y* = 0.4098× + 2597.1, *r* = 0.901, *df* = 7, *p* = 0.001) and spatial displacement (Figure 3D) (*y* = 0.0005× + 1.4745, *r* = 0.959, *df* = 7, *p* < 0.001). The effect of actual frequency on spatial displacement (*r* = 0.959) was significantly greater than the effect of space on frequency estimation (*r* = 0.593) (3A vs. 3B, difference of correlations = 0.366, Fisher r-to-z transformation, *z* = 2.17 one-tailed, *p* < 0.05). Correlation coefficients for PITCH→TIME (*r* = 0.90) and TIME→PITCH (*r* = 0.79) effects were not significantly different from one another.

Complementary effects were found using multiple regression analyses. Distance was significantly correlated with frequency judgments even when variance associated with each trial’s actual frequency was removed [ρ*r*(81) = 0.33; *df* = 80, *p* = 0.003], and duration was significantly correlated with frequency judgments even when variance associated with each trial’s actual frequency was removed [ρ*r*(81) = 0.45; *df* = 80, *p* < 0.001]. Frequency was significantly correlated with duration judgments even when variance associated with each trial’s actual duration was removed [ρ*r*(81) = 0.54; *p* < 0.001]; and with distance judgments even when variance associated with each trial’s actual distance was removed [ρ*r*(81) = 0.78; *p* < 0.001]. There was no effect of direction (left-moving vs. right moving trials).

Participants’ overall estimates of duration, spatial displacement, and pitch were accurate. The effects of actual duration on estimated duration (*y* = 187.04× + 2122 *r* = 0.94, *df* = 7, *p* < 0.001), actual frequency on estimated pitch (*Exp. 2A*: *y* = 0.2555× + 431.53 *r* = 0.95, *df* = 7, *p* < 0.001), actual spatial displacement on estimated displacement (*y* = 0.4874× + 0.6134 *r* = 0.99, *df* = 7, *p* < 0.001), and actual frequency on estimated pitch (*Exp. 2B*: 0.4425× + 306.19, *r* = 0.99, *df* = 7, *p* < 0.001) were all highly reliable but not significantly different from one another. Again, these results suggest that spatial, temporal, and pitch changes are no more or less “hard to perceive” (Casasanto, 2017) in the current procedure.

### Experiment 2 Discussion and Results Summary for Experiments 1 and 2

We predicted that if space is less relevant than time in the auditory modality then pitch should affect spatial judgments *more* than temporal judgments (PITCH→SPACE > PITCH→TIME), but that space should be *less* effective than time in influencing pitch judgments (SPACE→PITCH < TIME→PITCH). The significant asymmetry in the effects of pitch-on-space vs. space-on-pitch, together with an inspection of the *r* values (Figure 4B) is consistent with predictions based on the degree of modality-relevance of space, time, and pitch “in sound.” The pattern of results suggests that in the auditory modality, space is particularly sensitive to irrelevant information while being less effective in modulating other kinds of information.

Across Experiments 1 and 2 in terms of the strength and direction of the respective correlation, a domain’s relative level of modality-relevance was predictive of both how well it performed as an *agent*, or modulator of other domains (*r* = 0.96, Figure 4C), and as a *patient* when examining the extent that it was sensitive to modulation by other domains (*r* = –0.98, Figure 4D). These predictions run counter to those made by Conceptual Metaphor Theory, general patterns in language use, and a previous literature that often portrays time as fundamentally more abstract than space.

## General Discussion

An earlier study (Casasanto and Boroditsky, 2008) using visual stimuli found strong evidence for an asymmetrical relationship between space and time, such that the remembered size of a stimulus in space modulated recall for its duration, but not *vice versa*. In contrast, Experiment 1 having an analogous design but using auditory stimuli found that space and time are mutually contagious. Furthermore, as predicted by the privileged relation between auditory and temporal processing, the perceived duration of a stimulus had a larger effect on perceived spatial displacement than the reverse. In order to further investigate the relevance of space and time in the auditory modality, Experiment 2 examined the mutual effects of space, time, and pitch. We reasoned that if space is less modality-relevant than time in sound, space should be *more* easily contaminated by pitch, while being *less* effective in contaminating it. While time and pitch were shown to be mutually contagious, pitch affected estimates of space but not *vice versa*. Across Experiments 1 and 2, results suggest that the visual asymmetry between space and time does not generalize to other domains like audition, and that time is not fundamentally more abstract than space.

While the present results are suggestive of a perceptual asymmetry running opposite to that observed in the visual domain, strong claims regarding a deep embodied asymmetry between time and space in the auditory domain require further support. Nor should it be assumed that the presence of modality-specific asymmetries suggests those of equal strength (to those found in vision) in the opposite direction. Notably, the effect of spatial displacement on duration estimates was still strong in the auditory domain (*r* = 0.88). In Casasanto and Boroditsky’s (2008) study, actual duration had no discernable effect on spatial displacement judgments. Furthermore, although “in sound,” space appears to be less relevant than time, these results may not generalize to other scales, intervals, and ranges of time–space relations. And while the methods in the current auditory study attempted to mirror those of the original visual study, there are some differences. For example, whereas Casasanto and Boroditsky’s (2008) study used a relatively “active” task requiring participants to reproduce the spatial or temporal extent of the visual target with a mouse drag or click in “real” space, the current study used a relatively “passive” one in that participants responded to a playback sound, stopping it when it reached a certain duration or location in “virtual” space. The auditory reproduction task in the current study required that participants remain passive while the sound object moved through space and time to reach a certain location, duration, or frequency. However, the playback sounds were always the same: duration could not be used to judge distance, and distance could not be used to judge duration. Casasanto and Boroditsky’s study required dragging a mouse between mouse clicks on spatial trials or clicking a stationary mouse on time trials. This task additionally required participants to translate between a visual stimulus and a motoric response in analog space. Also, because it generally takes a longer time to travel a longer distance, despite orthogonalizing space and time in the target stimuli, duration and spatial displacement may have been correlated across participants’ reproduction responses, but only on space trials. Future studies could aim to use identical, modality- and domain-unbiased reproduction tasks, using both visual and auditory stimuli across a range of scales; although it should be noted that equating scales between distinct perceptual modalities at the level of psychophysics and phenomenology is never straightforward. That is, identical distances may not scale and behave identically across vision and sound.

Another limitation concerns the extent to which one can isolate and describe the mechanism for producing the pattern of results described here. The current experiments (and previous studies on which it is based) require participants to attend to a perceived location, duration, and/or frequency of an auditory stimulus before being tasked to reproduce one of these dimensions by responding to a later target sound. This means that participants were required to maintain information in working memory prior to making a response. Therefore, based on the current data, it is not possible to differentiate where cross-domain contamination occurs with respect to attention, perception, and memory. Moreover, an extensive psychophysics literature has shown that visual and auditory stimuli, along with temporal and spatial information, show differences with respect to how they are attended to and processed, both online and in working memory (Cohen et al., 2009; Protzner et al., 2009; Delogu et al., 2012; Thelen et al., 2015). The approach used here does not allow us to determine where or when contamination occurs, only that it does in the auditory domain in ways that are not predicted by previous theory. Future studies, in describing what aspects of a stimuli are more or less “modality-relevant” would do well to better ground such assertions in the experimental psychophysics literature. In fact, the current study should be considered an invitation to do so.

Still these results suggest that time is not necessarily or fundamentally more abstract than space, and that previously observed verbal and mental asymmetries of *representing time in terms of space* may at least be partially dependent on the human disposition to think visually. The general idea that visuospatial representations are central to how people talk and think is well established (Johnson-Laird, 1986; Talmy, 2000; Chatterjee, 2001; Tversky, 2005). In the context of previous research demonstrating a strong asymmetry for time–space relations, the results of the present study suggest something very important about the nature of those “embodied spatial representations” that appear to structure patterns in language and thought. That is, such representations are likely visuospatial in nature. It should be noted that the present results in no way refute those reported in Casasanto and Boroditsky’s (2008) study. Rather, our results suggest that the common understanding throughout the literature that *time is generally more abstract than space* may need to be revised or at least more consistently articulated. This should not come as a total surprise because “space” is itself a very abstract concept and, like “time,” cannot be directly seen, touched, or heard. The present data, and the notion of modality-relevance, suggest that what makes certain spatial or temporal *relations* more or less abstract (in the terms of Conceptual Metaphor Theory) are the sensory modalities in which those relations are preferentially processed or experienced. As such, the present results support a refined but intuitive view of embodied cognition that takes into account contributions of a particular sensory modality in processing the qualities of a stimulus. While space and time may both be very abstract according to such an understanding, relations between objects immersed in either substrate (whether seen or heard) may be more or less so depending on a range of species-specific and contextual variables.

For humans, “embodied spatial representations” important for structuring other forms of thought and language may be most accessible when they are visuospatial in nature. Because humans have a general visual bias in perception, communication, and neural organization, there may be a tendency for us to experience and understand space as relatively less abstract than time. But this does not mean that space is necessarily less abstract than time, or that other organisms experience space and time as we do. While it is famously difficult to imagine the quality of conscious experience in another organism (Nagel, 1974) perhaps it is the case that animals (like bats) which rely more on audition than vision to locate objects in a dynamic environment could be biased to understand time as less abstract than space (if they had opinions on such matters). This is merely to say, that what is experienced as “abstract” may be a function of an organism’s particular form of embodiment, rather than a set of formal ontological (metaphysical) relations.

A more tractable issue worth reconsidering concerns the question of why time is generally assumed to be more abstract than space in the first place. The argument may be based on the idea that time, as compared to space, cannot be “directly perceived” (Ornstein, 1969), or that we cannot “see or touch” time (Casasanto et al., 2010). Yet there are known, widely distributed, neural mechanisms specific to temporal processing, and little basis for the assumption that spatial relations are themselves perceived directly (Kranjec and Chatterjee, 2010). The experience of space and time both involve inherently relational processes, making the representation of both domains relatively abstract.

For example, processing locations between objects in an array using vision is arguably no more or less direct than processing rhythm in a sequence of beats using audition, with each requiring the representation of a number of abstract *relations* between objects or sounds. That is, there is no reason to think that we can directly “see” space any more than we can “hear” time. Nowhere is the dissociation between vision and spatial processing more apparent than in *simultanagnosia*, a neuropsychological condition in which patients are characteristically unable to perceive more than a single object despite having intact visual processing (Luria, 1959; Coslett and Chatterjee, 2003). Nonetheless, visuo-spatial and audio-temporal relations appear to be privileged. Privileged relations between particular sensory modalities and experiential domains may play some part in determining what we come to label abstract or concrete. Further research is needed to determine why some senses are subjectively felt to be more or less abstract than others, and the specific roles that spatial and temporal organization play in structuring our sensory experience.

## Author Contributions

AK, ML, and AC conceived and designed the experiments. ML performed the experiments. AK, ML, and AW analyzed the data. AK and AC wrote the manuscript.

## Conflict of Interest Statement

The authors declare that the research was conducted in the absence of any commercial or financial relationships that could be construed as a potential conflict of interest.
